# Optimisation and standardisation of a multiplex immunoassay of diverse *Plasmodium falciparum* antigens to assess changes in malaria transmission using sero-epidemiology

**DOI:** 10.12688/wellcomeopenres.14950.2

**Published:** 2020-04-23

**Authors:** Lindsey Wu, Tom Hall, Isaac Ssewanyana, Tate Oulton, Catriona Patterson, Hristina Vasileva, Susheel Singh, Muna Affara, Julia Mwesigwa, Simon Correa, Mamadou Bah, Umberto D'Alessandro, Nuno Sepúlveda, Chris Drakeley, Kevin K A Tetteh

**Affiliations:** 1 Department of Infection Biology, London School of Hygiene & Tropical Medicine, London, WC1E 7HT, UK; 2 Infectious Diseases Research Collaboration (IDRC), Kampala, P O. Box 7475, Uganda; 3 Department for Congenital Disorders, Statens Serum Institut, Copenhagen, Denmark; 4 Centre for Medical Parasitology at Department of International Health, University of Copenhagen, Copenhagen, Denmark; 5 Infectious Disease Epidemiology Department, Bernhard Nocht Institute for Tropical Medicine, Hamburg, 20359, Germany; 6 MRC Unit at the London School of Hygiene and Tropical Medicine, Fajara, The Gambia

**Keywords:** Plasmodium, malaria, antibodies, serum, serology, Luminex, MAGPIX

## Abstract

**Background:** Antibody responses have been used to characterise transmission and exposure history in malaria-endemic settings for over a decade. Such studies have typically been conducted on well-standardised enzyme-linked immunosorbent assays (ELISAs). However, recently developed quantitative suspension array technologies (qSAT) are now capable of high-throughput and multiplexed screening of up to hundreds of analytes at a time. This study presents a customised protocol for the Luminex MAGPIX ^©^ qSAT using a diverse set of malaria antigens. The aim is to develop a standardised assay for routine serological surveillance that is implementable across laboratories and epidemiological settings.

**Methods:** A panel of eight *Plasmodium falciparum *recombinant antigens, associated with long- and short-lived antibody responses, was designed for the Luminex MAGPIX ^©^ platform. The assay was optimised for key steps in the protocol: antigen-bead coupling concentration, buffer composition, serum sample dilution, and bead storage conditions. Quality control procedures and data normalisation methods were developed to address high-throughput assay processing.  Antigen-specific limits of quantification (LOQs) were also estimated using both in-house and WHO reference serum as positive controls.

**Results:** Antigen-specific bead coupling was optimised across five serum dilutions and two positive controls, resulting in concentrations operational within stable analytical ranges. Coupled beads were stable after storage at room temperature (22⁰C) for up to eight weeks. High sensitivity and specificity for distinguishing positive and negative controls at serum sample dilutions of 1:500 (AUC 0.94 95%CI 0.91-0.96) and 1:1000 (AUC 0.96 95%CI 0.94-0.98) were observed. LOQs were also successfully estimated for all analytes but varied by antigen and positive control.

**Conclusions:** This study demonstrates that developing a standardised malaria-specific qSAT protocol for a diverse set of antigens is achievable, though further optimisations may be required. Quality control and data standardisation methods may also be useful for future analysis of large sero-epidemiological surveys.

## Introduction

Until recently, the vast majority of malaria antibody studies have sought to understand the acquisition of protective immunity to inform vaccine development ^1, 2^. However, there is growing interest in identifying new serological markers of malaria exposure for epidemiological surveillance ^3– 5^. Rational selection of these markers for population-wide sero-profiling could enable the development of improved tools for monitoring changes in malaria transmission. Such tools have the potential to simultaneously characterise both historical and recent patterns in malaria exposure.

Accurately quantifying antibody dynamics in these contexts requires population-representative or frequently-sampled longitudinal datasets ^3, 6^. Sero-epidemiological studies of this scale have historically used enzyme-linked immunosorbent assays (ELISAs), which are easily standardised, widely available, and ideal for high-throughput analysis of a single antigen (or the combined response to multiple antigens). However, the limited dynamic range and need for relatively high blood volumes make ELISAs less efficient for evaluating multiple analyte-specific responses. Conversely, protein microarrays allow high-throughput analysis of hundreds to thousands of analytes per sample ^7, 8^, but are still prohibitively expensive and not easily accessible by national malaria control programmes (NMCPs) or laboratories.

Cytometric bead array (CBA) and quantitative suspension array technologies (qSATs), such as Luminex xMAP© (Luminex Corp, Austin TX), are now available as affordable mid- to high-throughput multiplexing platforms. These offer several advantages, including the simultaneous quantification of 50-500 proteins in a single well, the use of standard 96- or 384-well plates, and requiring as little as 5μl of plasma or serum ^9– 11^. These platforms have also been shown to measure a larger dynamic range of antibody responses compared to ELISA ^12, 13^.

Optimisation of CBA and qSAT platforms can be complex for antigen panels designed to capture a wide range of antibody dynamics. A key challenge is achieving a high degree of multiplexing while retaining differential responses across a diverse set of antigens ^14^. Additionally, standardised approaches for the epidemiological analysis of Luminex data are still in development. A number of recent studies have assessed cluster-level antibody responses based on Luminex data for malaria ^15– 18^ and other infectious diseases ^19, 20^, but there is still a paucity of data on appropriate methods for standardisation and interpretation across laboratories, sites, and antigens.

In this study, we developed a customised panel of *Plasmodium falciparum* ( *Pf)* recombinant antigens as serological markers of both historical and recent malaria exposure and optimised a protocol for the Luminex MAGPIX ^©^ qSAT platform. This includes five recently developed antigens previously validated in protein microarray studies for their association with recent malaria infection in Ugandan and Malian children ^3^. For epidemiological analysis, we present quality control procedures for high-throughput assay processing, data normalisation methods, and report estimates of antigen-specific limits of quantification (LOQs). The aim was to translate the development of a suite of markers for malaria exposure to a qSAT platform that is practical for epidemiological surveillance across laboratories and countries.

## Methods

**Figure 1.**
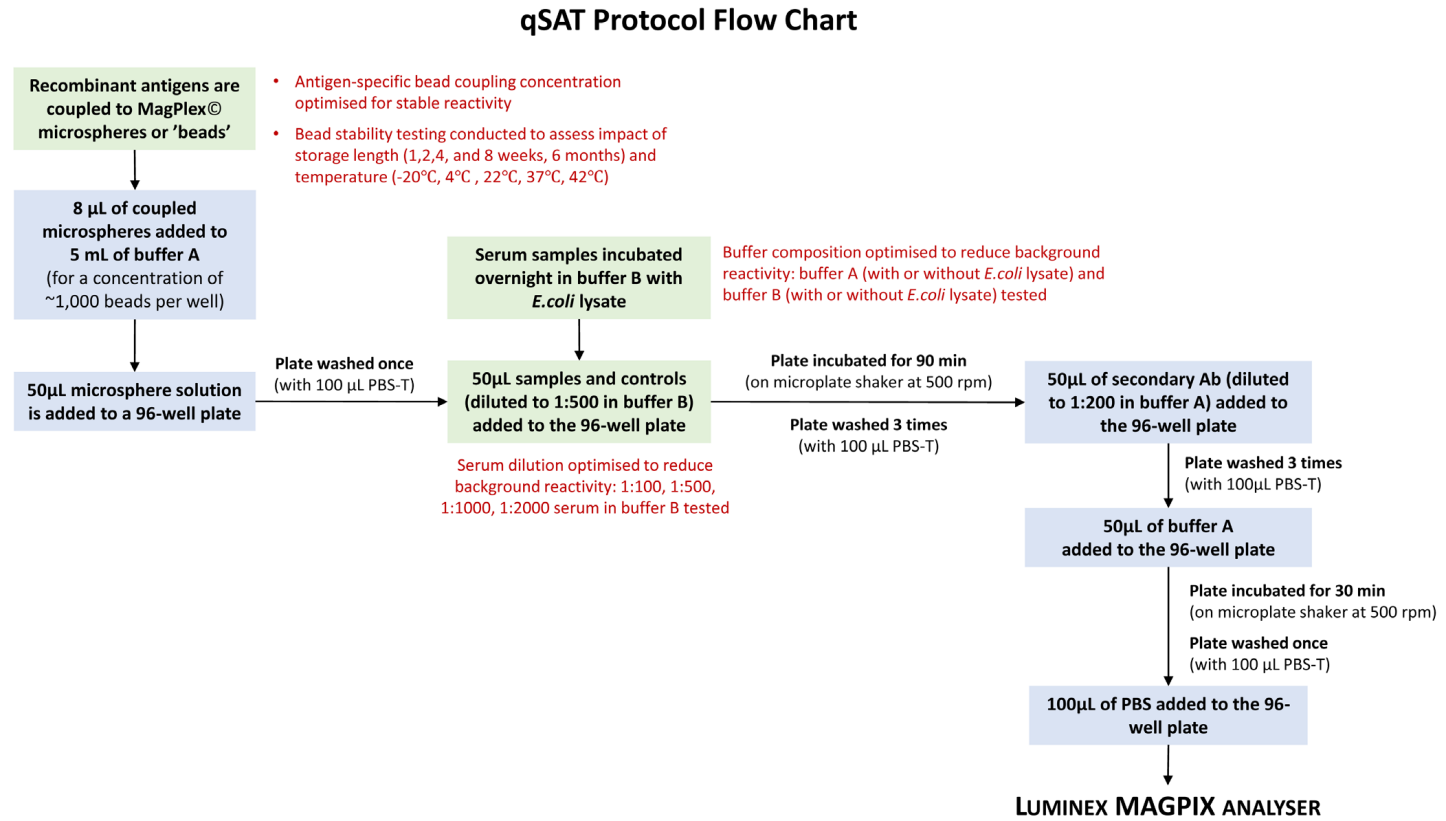
Scheme describing the qSAT assay protocol. Assay conditions tested for optimisation indicated in green boxes and red text.

Assay conditions were assessed and optimised for key steps in the protocol: antigen-bead coupling concentration, buffer composition to reduce non-specific reactivity, serum sample dilution, and the impact of storage length and temperature on bead stability ( Figure 1).

### Antigen selection and design

A multiplex panel was developed for the Luminex MAGPIX ^©^ suspension bead array containing eight erythrocytic *Pf* recombinant proteins ( Table 1). Antigens were selected from an initial screen of 856 candidates on an *in vitro* transcription and translation (IVTT) protein microarray assay based on their correlation with previous malaria infection in children ^3^. Each antigen was generated and expressed in *Escherichia coli* as glutathione S-transferase (GST)-tagged fusion proteins using methods as previously described by Herman *et al.* ^21^, Tetteh *et al.* ^22^, and Polley *et al.* ^23^. The exception to the panel was *Pf* AMA1, which was expressed in Pichia pastoris as a histidine-tagged protein ^24^. Protein purification was conducted by affinity chromatography (Glutathione Sepharose 4B (GE Healthcare Life Sciences) or HisPur Ni-NTA (Invitrogen) resins for GST and His tagged proteins, respectively), and the concentration, quality, and purity of the antigen yield was assessed using a Bradford assay and SDS-PAGE. Bacterial lysate was generated from the culture of untransformed *E.coli* and used in the preparation of assay buffers as non-specific protein to eliminate background reactivity to E.coli proteins ^3^.

**Table 1.** Summary of antigens in multiplex Luminex panel.

Gene ID	Antigen name	Allele	Expression	Location	Description
PF3D7_0930300	*Pf*MSP1 _19_^†^	Wellcome	GST	Merozoite surface	19kDa fragment of MSP1 molecule ^23^
PF3D7_1133400	*Pf*AMA1 ^†^	FVO	His _x6_	Sporozoite / Merozoite	Apical membrane antigen1 ^24^
PF3D7_1035300	*Pf*GLURP.R2	F32	N/A	Merozoite	Glutamate rich protein R2 ^25^
PF3D7_0532100	Etramp5.Ag1	3D7	GST	iRBC / PVM	Early transcribed membrane protein 5 ^26^
PF3D7_0423700	Etramp4.Ag2	3D7	GST	iRBC / PVM	Early transcribed membrane protein 4 ^27^
PF3D7_0402400	GEXP18	3D7	GST	Gametocytes	Gametocyte exported protein 18 ^3^
PF3D7_0501100.1	HSP40.Ag1	3D7	GST	iRBC / Gametocytes	Heat shock protein 40, type II ^3^
PF3D7_1002000	Hyp2	3D7	GST	iRBC / PVM	Plasmodium exported protein ^3^
**--**	GST	--	--	--	GST expression tag
**--**	TT	--	--	--	Tetanus Toxoid

^†^Conformational protein. iRBC, infected red blood cell; PVM, parasitophorous vacuole membrane; GST, glutathione S –transferase.

### Positive and negative controls for assay optimisation

For the optimisation of the assay protocol, several different positive controls were used and are summarised for each test condition ( Table 2). This included pooled serum from hyper-immune individuals in Tanzania (CP3), Uganda (PRISM), The Gambia (Brefet) as well as the WHO reference serum (NIBSC 10/198) ^28^. Individual plasma samples from European malaria-naive adults were used as negative controls.

**Table 2.** List of assay conditions tested.

Optimisation	Values tested	Samples
**Antigen concentration**	6-dilution serial protein titration 2 positive controls 5 serum sample dilutions (1:100, 1:200, 1:400, 1:800, 1:1600)	• Tanzanian pooled serum (CP3) • WHO reference serum (NIBSC 10/198)
**Buffer composition**	Buffer A • With *E.coli* lysate • Without *E.coli* lysate Buffer B • With *E.coli* lysate • Without *E.coli* lysate	• 40 Gambian individuals (Brefet) • 40 malaria naive European blood donors (PHE) Samples tested at serum dilution of 1:100
**Sample dilution**	1:100, 1:500, 1:1000, 1:2000	• Tanzanian pooled serum (CP3) • 2 sets of Ugandan pooled serum (Apac, PRISM) • Gambian pooled serum (Brefet) • 12 Ugandan individuals • 4 Gambian individuals • 20 malaria naïve European blood donors (PHE) Samples incubated in buffer B with *E.coli*
**Microsphere storage**		
• Length of time	<4 weeks storage at 4°C 6 months storage at 4°C	• Tanzanian pooled serum (CP3) • 2 sets of Ugandan pooled serum (Apac, PRISM) • Gambian pooled serum (Brefet) • 4 malaria naive European blood donors (PHE) Samples tested at serum dilution of 1:100 and incubated in buffer B with *E.coli*
• Temperature	1-8 weeks storage at: -20°C, 22°C, 37°C and 42°C	• Tanzanian pooled serum (CP3) Samples tested at serum dilution of 1:100 and incubated in buffer B with *E.coli*

### Microsphere coupling, buffer, and sample dilution optimisation

Recombinant antigens were coupled to MagPlex© COOH-microspheres or ‘beads’ (Luminex Corp., Austin TX) following the protocol described by the Luminex Corporation ^29^. Optimal coating concentrations for each antigen were tested using a six-point serial titration of protein. Starting dilutions were determined by the known immunogenicity range of each antigen ( Table 3). Titrations were tested under a series of conditions to assess the variability across 1) two different positive pools (CP3 and WHO NIBSC 10/198) and 2) five serum sample dilutions (1:100, 1:200, 1:400, 1:800, and 1:1600). All samples were incubated overnight in buffer B with *E. coli* lysate. The antigen titration at mid-point of the median fluorescence intensity (MFI) dose-response curve (EC _50_ or MFI _50_) was calculated for each positive pool and serum dilution condition (15 in total for each antigen), using Equation 1 described further below. The median titration across all conditions was selected as the optimal coupling concentration for each antigen and used for large volume bead coupling. Beads were then re-suspended in 1 mL of storage buffer (1x phosphate buffered saline (PBS pH 7.2), 0.05% Tween, 0.5% bovine serum albumin (BSA), 0.02% sodium azide, 0.02% Pefabloc (Sigma)) and stored at 4°C until further use.

**Table 3.** Antigen coupling concentration titrations. Optimal range and final antigen concentration in bold.

	Antigen titration (µg/mL)							
Antigen	1	2	3	4	5	6	Final coupling concentration (µg/mL)	Final coupling concentration (ng/5000 beads)
*Pf*MSP1 _19_	800.00	**100.00**	**12.50**	1.563	0.195	0.024	**51.5**	**20.6**
*Pf*AMA1	600.00	75.00	**9.375**	**1.172**	0.147	0.018	**3.85**	**1.54**
*Pf*GLURP.R2	300.00	37.50	4.688	0.586	**0.073**	**0.009**	**0.042**	**0.017**
Etramp5.Ag1	**1000.00**	**125.00**	15.625	1.953	0.244	0.031	**243**	**97.2**
GEXP18	**750.00**	**93.75**	11.719	1.465	0.183	0.023	**618**	**247.2**
HSP40.Ag1	800.00	**100.00**	**12.50**	1.563	0.195	0.024	**91.5**	**36.6**
Etramp4.Ag2	1000.00	**125.00**	**15.625**	1.953	0.244	0.031	**32.5**	**13.0**
Hyp2	**350.00**	**43.75**	5.469	0.688	0.085	0.011	**197**	**78.8**

Antigen reactivity was tested for sensitivity to buffer composition and serum sample dilution. To reduce non-specific background reactivity for antigens expressed in *E. coli*, factorial testing of the two buffer solutions (buffer A - 1xPBS, 0.05% Tween, 0.5% BSA, 0.02% sodium azide; and buffer B - 1xPBS, 0.05% Tween, 0.5% BSA, 0.02% sodium azide, 0.1% casein, 0.5% polyvinyl alcohol (PVA), 0.5% polyvinyl pyrrolidone (PVP)) was conducted. Both buffers were tested with or without supplementation of *E. coli* lysate (added at 15.25 μg/ml), resulting in a total of four different buffer compositions. Positive control samples were 40 individuals from Brefet, The Gambia and negative controls were 40 malaria-naïve European individuals (all samples tested at 1:100 dilution). The effects of buffers A and B - with or without *E. coli -* on MFI values of both positive and negative samples were assessed using linear regression with an interaction term to test for potentially synergistic effects on background reactivity by adding *E. coli* to buffer B.

A range of serum concentrations was also tested (1:100, 1:500, 1:1000 and 1:2000) using samples from 20 individuals from endemic regions in Uganda, Tanzania, and The Gambia as positive controls and 20 malaria naïve European individuals as negative controls. All samples were incubated overnight in buffer B with *E. coli* lysate. Optimal serum sample dilutions were selected based on cross-validated Area Under the Receiver Operating Characteristics Curve (AUC) values, calculated from the sensitivity and specificity of continuous MFI values for predicting a positive or negative control using the *ci.cvAUC* function in the ‘ cvAUC’ package version 1.1.0 (R version 3.5.1).

### Stability and reproducibility testing

To evaluate the impact of storage temperature, accelerated stability testing was conducted using aliquots of antigen-coupled beads stored for 1–8 weeks at -20°C, 22°C (room temperature), 37°C and 42°C. Antigen-coupled beads were incrementally added to each storage temperature at intervals over an 8-week period, such that total storage time ranged from 1, 2, 4, and 8 weeks total. All the antigen-coupled beads were run simultaneously at the end of eight weeks with a single positive control titration, avoiding the need to adjust for random effects due to week or positive control batch in subsequent regression analysis. Beads were assayed using a five-point serial dilution titration and the CP3 Tanzanian positive pool (described above) at a 1:100 dilution in buffer B with *E. coli* lysate. The effect of each storage condition was assessed with multivariate linear regression to estimate change in MFI over time, adjusted for storage temperature and sample concentration and allowing for pairwise interaction between all covariates (storage temperature, storage time and sample concentration).

To assess the impact of long-term storage on stability and reproducibility of results, two sets of antigen-coupled beads were assessed; one batch stored for 6 months at 4°C and another batch stored for less than 4 weeks at 4°C. Pooled serum of hyper-immune individuals from Tanzania, Uganda, and The Gambia were each tested in triplicate with beads from each storage condition (less than 4 weeks and 6 months). Change in logMFI at 6 months compared to less than 4 weeks storage was assessed using linear regression, adjusting for antigen and allowing for random effects by sample and replicate.

### Final qSAT assay procedure

General assay procedures were as follows and illustrated in Figure 1. First, an initial mixture containing 8 μl of each set of antigen-coupled microspheres and 5 ml of buffer A was prepared, yielding approximately 1,000 beads per region per well (based on optimal conditions reported in previous studies ^5, 13^). Next, 50 μl of this combined microsphere mixture was added to a 96-well flat bottom plate (BioPlex Pro™, Bio-Rad Laboratories, UK) and washed once by placing the assay plate onto a magnetic plate separator (Bio-Plex®, Bio-Rad Laboratories, UK) and pausing for 2 minutes. Plates were then inverted forcefully to remove the liquid and 100 μl of PBS-T (1xPBS, 0.05% Tween-20) added to each well. Next, 50 µl of samples and controls were added to the plate and incubated in the dark at room temperature (RT) on a microplate shaker at 500 rpm for 90 minutes. Following three washes, 50 μl of fluorescent secondary antibody (Jackson Immuno 109-116-098: Goat anti-human Fcy-fragment specific IgG conjugated to R-Phycoerythrin (R-PE)), diluted to a 1:200 dilution with buffer A, was added to all wells and incubated for 90 minutes in the dark at RT at 500 rpm. After a further three washes, the plate was incubated in 50 μl of buffer A for 30 minutes. Plates received an additional wash and, after a final addition of 100 μl 1xPBS, were read using the Luminex MAGPIX ^©^ analyser. At least 50 beads per analyte were acquired per sample and MFI data were used for analysis.

This protocol is used to screen samples from regions that may be co-endemic for both schistosomiasis and malaria, potentially causing non-malaria reactivity against GST-tagged fusion proteins. Therefore, GST-coupled beads were included to quantify any GST-specific immunoglobulin (IgG) responses, which can be subtracted from total MFI against GST-tagged fusion proteins to better reflect malaria-specific IgG responses. Beads coupled with tetanus toxoid vaccine protein (TT) were also included as an internal assay quality control, given that measurable MFI values are expected for positive controls from regions where tetanus vaccine coverage is high. All data reported and analysed are in units of background subtracted MFI (Net MFI - blank wells).

### Study samples used to develop standardisation methods

Samples used to validate data normalisation were based on all-age cross-sectional surveys conducted in July 2013 and December 2013 in two villages in the West Coast Region and two villages in the Upper River Region (N=1,813) of The Gambia ^30^. Samples were eluted from a 6-mm dried blood spot (DBS) punch, corresponding to 4 μl of whole blood, and shaken overnight at room temperature in 200 μl of elution buffer containing 1xPBS, 0.05% sodium azide and 0.05% Tween-20, yielding an initial 1:50 sample dilution. At least 1 day prior to assay processing, samples were further diluted to a final 1:500 dilution using 10 μl of the 1:50 pre-dilution sample and 90 μl of blocking buffer B with *E.coli* extract to prevent non-specific binding. Negative and positive controls were also incubated one day prior in buffer B with *E. coli*, with negative controls prepared at a 1:500 dilution and Gambian pooled positive controls in a 6-point 5-fold serial dilution (1:10 – 1:31,250). Two wells on each plate containing only antigen-coupled beads and buffer B, but absent of any human serum, were included to measure background signal. A pool of 22 serum samples from malaria hyper-immune individuals in Upper River Region, The Gambia were used as a positive control, and plasma from 10 European malaria-naive adults were used as negative controls.

### Estimating antigen-specific limits of quantification

To estimate the lower limit of quantification (LLOQ) and higher limit of quantification (HLOQ) for each antigen, two positive controls were tested (WHO NIBSC 10/198 and Tanzanian pooled positives (CP3)) using a 16-point 3-fold serial dilution, starting at 1:2. For each antigen and positive control, standard curves were fitted with Equation 1. The LLOQ MFI was defined as the MFI value where the upper 95%CI of the MFI _min_ parameter estimate is equivalent to the lower 95%CI of the standard curve estimate, and the HLOQ MFI where the lower 95%CI of the MFI _max_ parameter estimate is equivalent to the upper 95%CI of the standard curve estimate ^31^.

### Quality control

For quality control of samples from The Gambia, Levey-Jennings charts ^15^ were used to plot the mean MFI values of three concentrations from the positive control standard curve (high, 1:10; medium, 1:50; and low, 1:250) as well as the background values for each plate. The acceptable range of MFI values for inclusion in data analysis was defined as the mean ± two standard deviations of a subset of ten reference plates (selected based on the quality and consistency of their standard curve values). Plates with MFI values outside this range for at least two standard curve dilutions and at least three antigens were rejected and repeated. Assays were processed in-country using beads transported from London. To assess the potential impact of interruption to the cold chain on bead stability, points on the Levey-Jennings plots were also ordered by date of plate processing and linear regression used to test for potential changes over time.

### Immunoassay data normalisation

To account for observed between plate variation in positive control standard curves, data were adjusted using a loess normalisation method ^32^. This method was tested using cross-sectional samples from The Gambia, as described above. First, positive control standard curves for each plate of antibody concentrations versus MFIs were fitted using a 4-parameter logistic equation ^31, 33, 34^:

$$MFI\;=\;MFI_{max}\;+\;\frac{\left(MFI_{min}\;–\;MFI_{max}\right)}{\left[1+{\left(\frac{\mathrm{dilution}}{MFI_{50}}\right)}^{slope}\right]}\;(\mathrm{Equation}1)$$

where MFI _max_ is the upper asymptote or maximum MFI response of the standard curve, MFI _min_ is the lower asymptote or minimum MFI response of the standard curve, MFI _50_ is 50% of MFI _max_, *dilution* is positive control serum sample serial dilution, and *slope* is the Hill coefficient or slope factor of the dose-response curve. EC _50_ is the concentration or dilution that corresponds to MFI _50_.

Next, ten reference plates from the study were selected based on the quality and consistency of their standard curve fits. For each antigen, a composite standard curve was computed by calculating the mean MFI values for the reference plates for 100 dilutions between the highest and lowest dilution on the standard curve. For each plate, the plate-to-reference standard curve MFI difference (∆MFI) was calculated for these 100 concentration points and a loess regression fit to ∆MFI as a function of mean MFI. The raw MFI data for all samples on the plate were then adjusted by the predicted ∆MFI based on the loess regression fit. Data were not corrected for background signal given that the between plate variation was already accounted for in the loess normalisation and all background MFIs were below 30 and therefore negligible.

### Ethical approval

Written informed consent was obtained for all study participants. Ethical approval for the use of the Tanzanian samples was obtained from the institutional review boards of the National Institute of Medical Research of Tanzania, Kilimanjaro Christian Medical Centre (KCMC), and the London School of Hygiene & Tropical Medicine (LSHTM). For the Uganda (PRISM) samples, ethical approval was obtained from the Makerere University School of Medicine Research and Ethics Committee (REC REF 2011-203), Uganda National Council for Science and Technology (HS 1074), LSHTM Ethics Review Committee (Reference 6012) and the University of California, San Francisco on Human Research (Reference 027911). Finally, for the collection of the Gambian samples ethical approval was granted by the Scientific Coordinating Committee of the Medical Research Council (MRC) Laboratories in The Gambia and by the Joint MRC/Gambian Government Ethical Committee.

## Results

### Antigen to microsphere coupling

**Figure 2.**
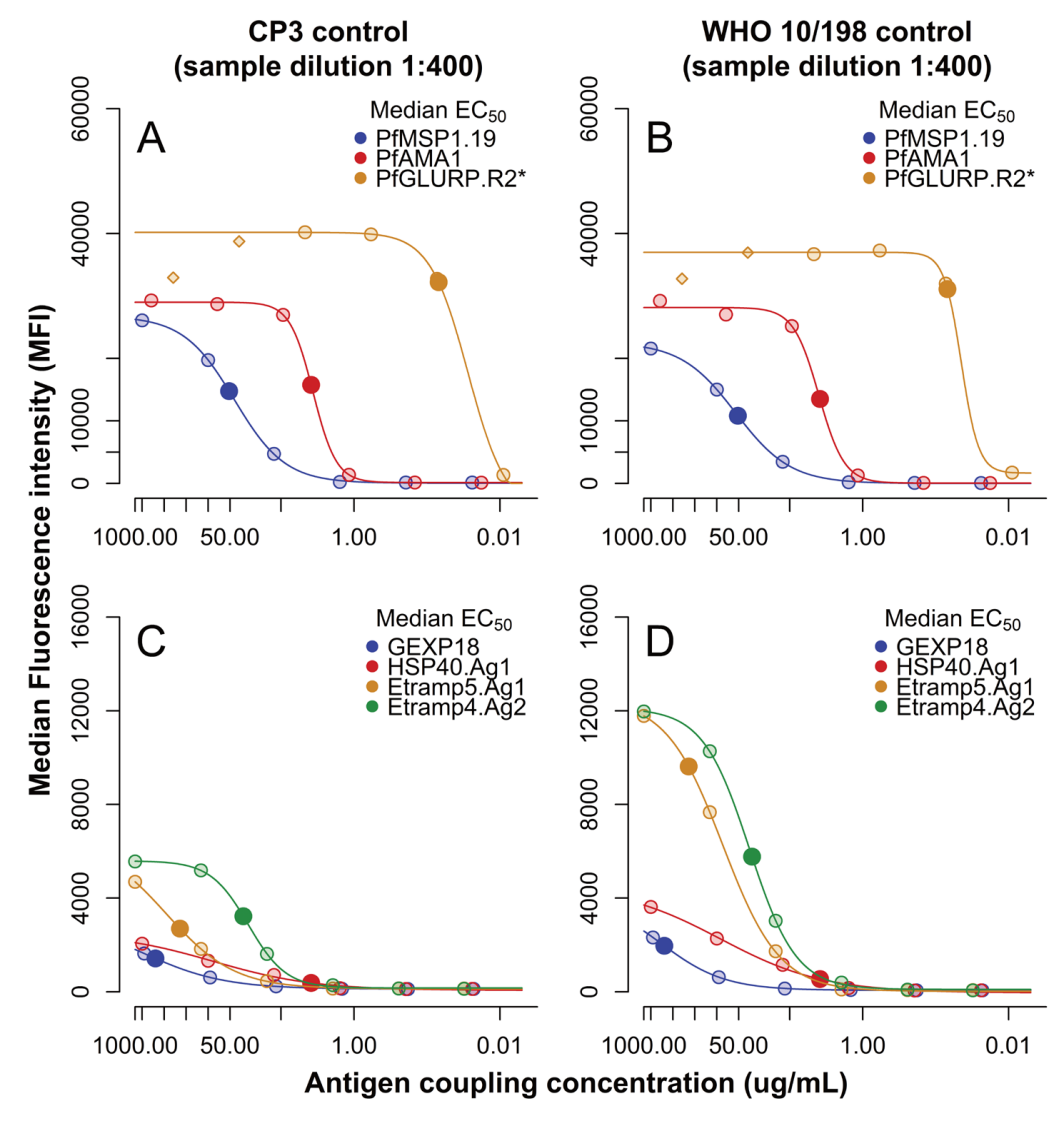
Titration of antigen concentration for microsphere coupling using two positive controls at 1:400 serum dilution. Antigens with maximum MFI values between 20,000 and 50,000 ( *Pf*MSP1 _19_, *Pf*AMA1 and *Pf*GLURP.R2) shown in ( **A**) and ( **B**) and between 1,000 and 20,000 (Etramp5.Ag1, GEXP18, HSP40.Ag1, Etramp4.Ag2, Hyp2) in ( **C**) and ( **D**). Coupled microspheres were tested on two positive controls: CP3 (left) and WHO reference 10/198 (right). Optimal antigen coupling concentration (median EC _50_ across all sample dilutions and positive controls) are indicated as solid filled circles. *For *Pf*GLURP.R2, the two highest antigen concentrations (shown as triangles) were not used to fit standard curves to exclude the influence of prozone effect.

Optimal protein concentration for microsphere coupling, as determined by the titration with the MFI value closest to EC _50_ of the dose-response curve, varied by antigen and ranged from as low as 0.017 ng/5000 beads for *Pf*GLURP.R2 to 618 ng/5000 beads for GEXP18 ( Figure 2, Table 3), depending on the immunogenicity of the recombinant protein. While some variation in estimated EC _50_ values for each antigen was observed between sample dilutions and positive controls (Supplementary Table S1), selecting the median EC _50_ across all conditions as the optimal antigen-coupling concentration translated to MFI values on the linear portion of the dose-response curve, resulting in antibody responses measurable within a stable analytical range (Figure S1–Figure S3). A sigmoidal curve could be fit to the data for all antigens except Hyp2, where the midpoint between the two lowest titrations was selected as the optimal antigen concentration instead of the EC _50_ ( Table 3, Supplementary Table S1).

### Bead storage stability

**Figure 3.**
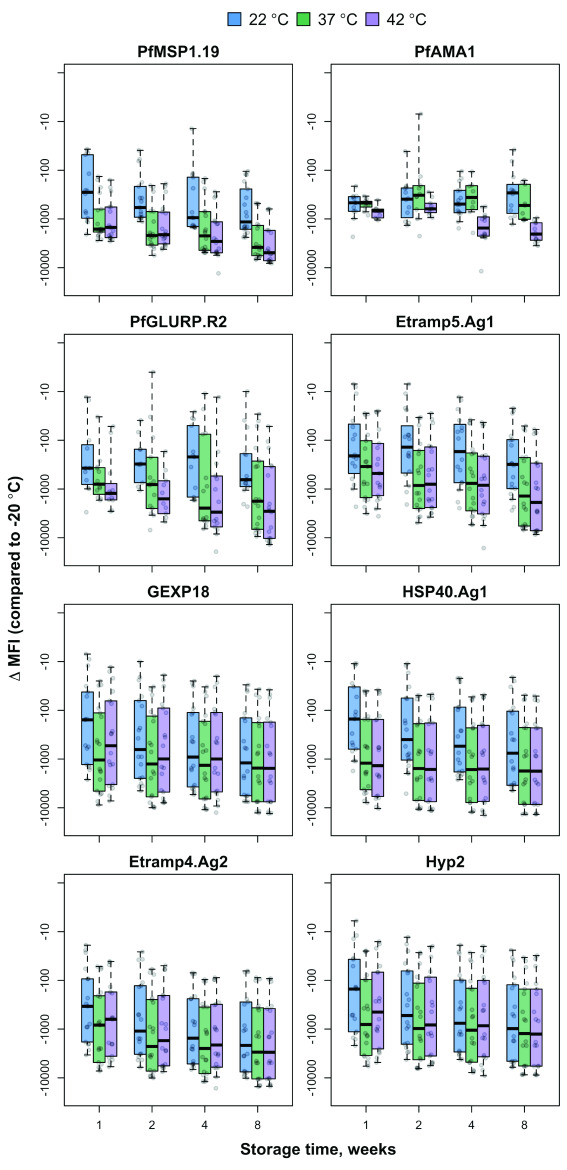
Bead stability by temperature, storage time and dilution factor. Difference in the median fluorescence intensity (∆MFI), of antigen-coupled beads stored at 22°C, 37°C and 42°C (compared to reference storage temperature of -20°C) after 1, 2, 4 and 8 weeks, and tested at six different positive control sample dilutions. Boxplots are based on data across all serum sample dilutions with median and interquartile range shown at each time point.

The effect of storage temperature on MFI signal over time varied by antigen, based on multivariate linear regression of change in MFI by storage week adjusted for temperature and serum sample dilution ( Figure 3, Supplementary Table S2 and Supplementary Table S3). Compared to storage at –20°C, all tested temperatures (22°C, 37°C, and 42°C) were associated with a decrease of over 1,000 MFI per week of storage for GEXP18, HSP40.Ag1, Etramp4.Ag2 and Hyp2. For Etramp5.Ag1, *Pf*MSP1 _19_ and *Pf*GLURP.R2, degradation of more than 1,000 MFI per week was only observed at storage temperatures of 37°C and 42°C. No significant decreases in MFI signal were observed for *Pf*AMA1 at any storage temperature. There was no significant degradation in signal between samples tested with beads stored for 6 months at 4°C compared to less than 4 weeks at 4°C, based on linear regression of logMFI with respect to time, after adjusting for antigen and allowing for random effects for sample and replicate.

### Buffer composition

**Figure 4.**
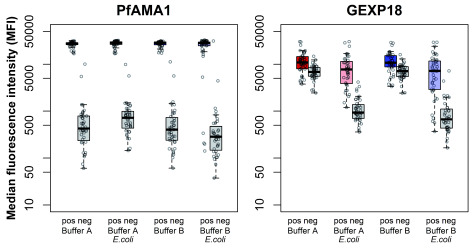
Median fluorescence intensity (MFI) for *Pf*AMA1 and GEXP19 of positive and negative samples for each buffer composition. Buffer compositions tested include buffer A (red), buffer A with *E. coli* lysate (pink), buffer B (blue), and buffer B with *E. coli* lysate (light blue). MFI for positive samples shown in colour (left) and corresponding negatives samples in grey (right) for each buffer composition.

In the absence of clarified *E. coli* lysate as a blocking agent against non-specific antibody binding to bacterial proteins, no significant differences in mean MFI were observed between the buffer compositions for both *Pf*AMA1 and GEXP18 ( Figure 4, Supplementary Table S4). The addition of the bacterial lysate was not associated with significant differences in MFI for yeast-produced *Pf*AMA1. However, for GEXP18, the addition of the bacterial lysate resulted in a significant reduction in non-specific binding for both malaria endemic (-3,203.50 MFI, p=0.035) and malaria naïve samples (-5,857.30 MFI, p<0.001). The combination of buffer B and *E. coli* lysate did not have a synergistic reduction on background reactivity.

### Serum sample dilution

Across all antigens tested, compared to serum dilution of 1:100, reductions in MFI values for negative controls were observed for sample serum dilutions of 1:500 (-911 MFI, p<0.001) and 1:1000 (-1,053, p < 0.001). These same serum dilutions maintained higher positive control values compared to a serum dilution of 1:2000 (Supplementary Figure S7). After adjusting for antigen, positive controls had an increased signal of 5,986 MFI (p<0.001) at a serum dilution at 1:500 and 3,006 MFI (p<0.001) at a serum dilution of 1:1000 compared to the average MFI signal at 1:2000. Higher sensitivity and specificity values were observed for serum dilutions 1:500 (AUC 0.94 95%CI 0.91-0.96) and 1:1000 (AUC 0.96 95%CI 0.94-0.98) compared to serum dilutions 1:100 (AUC 0.89 95%CI 0.86-0.93) and 1:2000 (AUC 0.91 95%CI 0.89-0.95), based on continuous MFI values across all antigens.

### Limits of quantification

**Figure 5.**
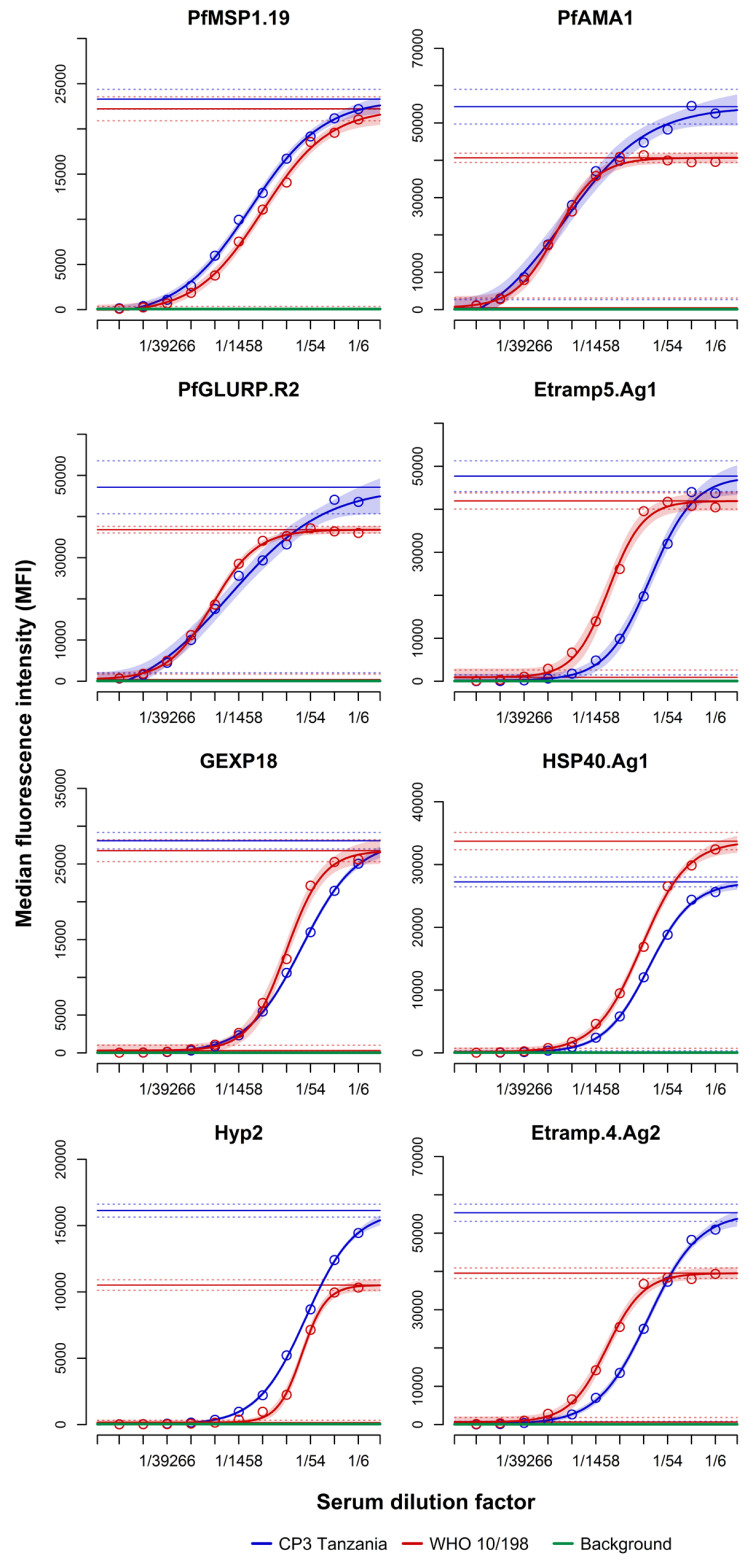
Limits of quantification. Median fluorescence intensity (MFI) values are shown for pooled Tanzanian hyper-immune serum CP3 (blue) and WHO reference serum (red) in a 12-point serial dilution. Horizontal lines represent the mean and 95% confidence intervals of the upper and lower asymptotes of the sigmoidal curve for CP3 (blue), WHO (red), and the mean background MFI in green.

The limits of quantification of the assay differed by antigen and positive control used ( Figure 5, Table 4). The highest HLOQs estimated were for *Pf*AMA1, *Pf*GLURP.R2, Etramp5.Ag1 and Etramp4.Ag2 (greater than 40,000 MFI using the CP3 Tanzanian positive control and greater than 30,000 MFI using the WHO reference standard). Estimated HLOQs for *Pf*MSP1 _19_, GEXP18, and HSP40.Ag1 ranged between 20,859 and 32,135 MFI across both positive controls, while the lowest estimated HLOQ was for Hyp2 (below 20,000 MFI for both controls). The lowest estimated LLOQs were for *Pf*MSP1 _19_, GEXP18, Hyp2, HSP40.Ag1 (below 500 MFI using the CP3 positive control), while estimated LLOQs for *Pf*AMA1, *Pf*GLURP.R2, Etramp5.Ag1, and Etramp4.Ag2 ranged 926–3,439 MFI across both positive controls tested.

**Table 4.** Limits of quantification at 1:400 serum sample dilution. Units in median fluorescence intensity.

	CP3		WHO	
	LLOQ	HLOQ	LLOQ	HLOQ
*Pf*MSP1 _19_	66	22,093	399	20,859
*Pf*AMA1	3,256	49,612	3,439	39,205
*Pf*GLURP.R2	2,533	40,691	1,754	35,913
Etramp5.Ag1	1,480	43,994	2,944	39,794
GEXP18	258	26,962	1,151	25,048
HSP40.Ag1	418	26,273	822	32,135
Etramp4.Ag2	926	52,967	1,912	38,082
Hyp2	132	15,570	379	10,042

Based on the CP3 positive control, the dynamic range was larger compared to the WHO reference control (e.g., both a lower LLOQ and higher HLOQ) for nearly all antigens including *Pf*MSP1 _19_, *Pf*AMA1, Etramp5.Ag1, Etramp4.Ag2, GEXP18, and Hyp2. *Pf*GLURP.R2 showed a higher HLOQ based on Tanzanian positive controls compared to the WHO reference standard, but also had a higher LLOQ. On the other hand, HSP40.Ag1 showed a lower LLOQ based on CP3 compared to the WHO reference, but also had a lower HLOQ.

### Quality control

A total of 7,868 blood samples from The Gambia cross-sectional study were processed (96×96-well plates). Out of 96 plates, 3 fell outside the acceptable range of MFI values and were repeated. Degradation of MFI signal after exposure of antigen coupled beads to storage conditions above room temperature was also observed over a period of two months of sample processing, based on linear regression of MFI by date of plate processing (Supplementary Figure S8). For positive controls run at a dilution of 1:250, mean MFI for *Pf*AMA1 at the start of sample processing was 24,480 and decreased at a rate of 61 MFI per plate (p=0.0104) over the course of two months. For GEXP18, mean MFI was 8,209 at the start of sample processing and declined at a rate of 31 MFI per plate (p=0.007) over the same period.

### Data normalisation

**Figure 6.**
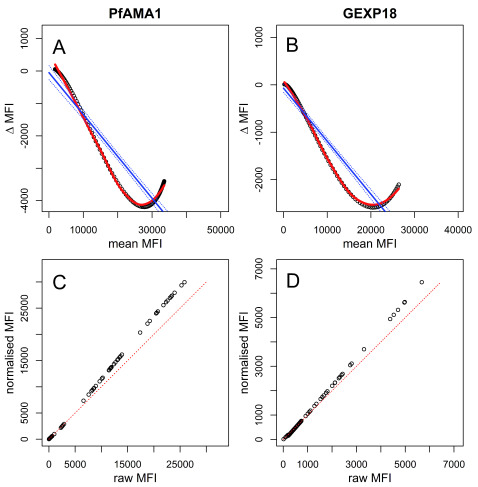
Loess normalisation for *Pf*AMA1 and GEXP18. Loess normalisation of antigens *Pf*AMA1 and GEXP18 is illustrated for one example plate based on cross-sectional samples from The Gambia. Panels **A** and **B** show the loess fit (red) and linear fit (blue) of ∆ MFI on the y-axis (the difference in unadjusted MFI of the standard curve of a single plate and the mean MFI of all standard curves from ten reference plates) and mean MFI on the x-axis. Panels **C** and **D** show the raw MFI values of individual samples on the x-axis versus normalised MFI values on the y-axis, and the equality line is shown diagonally in red.

Proportional differences in plate-specific MFI values compared with mean MFI values of reference plates (indicated as ∆MFI in Figure 6) were highly dependent on the MFI range ( Figure 6, Supplementary Figures S9-11). In other words, MFIs in the higher end of responses could show larger between-plate variations than MFIs in the lower end of responses (or vice versa) and may not be easily adjusted for using one proportional factor across the full range of MFI values- the method typically used for normalising ELISA-based data. The extent of the variations differed by plate and antigen. Therefore, the loess normalisation allowed for raw data to be adjusted and weighted according to MFI range-specific differences ( Figure 6, Supplementary Figures S9-11). Additionally, loess regression provided a better fit to the ∆MFI versus mean MFI data compared to linear regression, indicating that it may provide better adjustment values for plate-specific normalisation.

## Discussion

This study builds on previous work optimising the Luminex® qSAT as a multiplex platform of *Plasmodium* antigens. To develop a panel of serological markers for the characterisation of both historical and recent malaria exposure, the methods tested here aimed to standardise a protocol that could consistently measure a large range of antibody responses. This included the optimisation of antigen-to-bead coupling concentrations, testing of coupled-bead stability at a range of temperatures reflective of variable storage conditions across laboratories, as well as buffer composition and serum sample dilution to minimise non-malaria specific background reactivity.

Results showed that storage of antigen-coupled beads at temperatures below 37°C, and ideally at room temperature or lower, minimised degradation of MFI signal over an 8-week period for all antigens. Some antigens, such as *Pf*AMA1, exhibited more stability than others, where no significant degradation in MFI signal was observed over an eight-week period even at temperatures up to 42°C. Storage at room temperature also affected the bead stability for GEXP18, HSP40.Ag1, Etramp4.Ag2 and Hyp2. This is consistent with previous studies reporting that stability of beads is antigen-specific. This suggests that additional formulations to improve bead stability, such as lyophilisation in the presence of stabilisers, could be explored to minimise the impact on assay results. This is particularly critical when reagents are subject to transport or storage conditions with a high risk of interruption to the transport cold chain. Temperatures of 22°C and above were tested in this study to assess the potential impact of disrupted cold chain during shipping to or long-term storage in laboratories in low- and middle-income settings. Further work should also investigate bead stability during long-term storage at 4°C, the standard storage condition for most research groups using Luminex platforms.

Optimal antigen-to-bead coupling concentrations were determined for eight *Pf* antigens, including five markers of recent malaria exposure ^3^. These concentrations were selected to cover a range a positive controls and serum sample dilutions allowing for generalisability across multiple study conditions. Previous studies have found that bead coupling concentration is the most consistent factor influencing assay variability. Failing to control the density of antigens on the microsphere surface may result in either sub-optimal coating and low reactivity or over-coating and subsequent precipitation or aggregation of beads on the bottom of the plate, impairing surface suspension antibody binding ^13^. The degree to which this occurs and the tendency for non-uniform protein aggregation may also be antigen-specific, depending on the physical characteristics of protein structure. New coupling methods are currently in development, including biotinylated microspheres that allow coupling to specific antigen regions for more consistent protein orientation. Future work should validate the analytical reproducibility of these coupling concentrations by monitoring the consistency in MFI signal between bead sets and recombinant protein batches. This may also identify antigens that may be better suited on either bead-based or microarray platforms. Given that protocols are being developed for high-throughput processing, the selection of optimal bead coupling concentrations should ideally ensure a consistent analytical signal across bead sets (i.e., close to the EC _50_ point on the standard curve), while minimising the amount of antigen needed to achieve cost and volume efficiencies over time. If these analytical and operational challenges can be met across a diverse set of antigens, there may be strong potential for including these analytes on larger multi-disease Luminex panels.

The inclusion of *E. coli* lysate in the sample incubation buffer was found to be an effective blocking agent, particularly against non-specific antibody binding to antigens expressed in *E. coli* by significantly reducing the MFI signal in negative samples. The impact of the addition of the *E. coli* lysate is suggestive of co-purification of *E. coli* proteins, for which there was little evidence by ELISA but are likely only detected on the Luminex platform due to the higher dynamic range. Moving forward, the inclusion of additional chromatography separation techniques would minimise the presence of any co-purifying proteins and potentially negate the need for the *E. coli* lysate additive. Serum sample concentration was also optimal at both 1:500 and 1:1000 to reduce non-specific background reactivity in negative samples while retaining a measurable degree of reactivity in positive samples. These serum dilutions had higher sensitivity and specificity for identifying positive control sera compared to dilutions of 1:100 and 1:2000. Several other assay conditions have been validated in previous studies. This includes the testing of 1,000 compared to 2,000 beads per well ^5, 13^, processing samples in duplicate ^5, 14^, the use of plasma compared to DBS ^35^, and the comparison of single-plex with multiplex platforms ^5, 35^. Based on these studies, the protocol presented here uses 1,000 beads as an optimal baseline condition. Additionally, processing samples in duplicate, the use of plasma, and single-plex platforms did not significantly improve assay results. Therefore, optimal conditions confirmed from previous studies were incorporated into this protocol but not investigated further.

This study also reports antigen-specific limits of quantification (LLOQs and HLOQs) for several new *Pf* recombinant proteins, where the dynamic range varied between antigens and positive controls used. The WHO reference standard was found to have a narrower dynamic range compared to in-house positive control sera, which has also been observed in previous studies using the WHO positive control ^36^. This is likely due to the selection of sera for this reference standard based on small number of antigens primarily associated with long-lived antibody responses ( *Pf*MSP1 _19_, *Pf*AMA1, and *Pf*CSP) and from only one geographical location (Kenya). Therefore, it may not be well suited for capturing short-lived antibody responses in populations from other endemic settings. Future work should consider how reference standards can be further improved to maximise the measurable range for a larger selection of *Pf* antigens and across different geographical regions and also determine the LOQs across a range of sample serum dilutions currently being used.

Several challenges remain for the further development of multiplex serological platforms for malaria surveillance. First, many Luminex-based malaria studies currently use antibody concentrations estimated from the positive control standard curve for epidemiological analysis. As already reported in previous studies, assay conditions can affect the fit of the standard curve used to normalise the data to arbitrary concentration units. This potentially results in large deviations in concentration estimates. MFI responses measured independently from a standard curve might reflect true variation, while normalisation methods that convert to relative concentration values will be more influenced by the precision of the statistical fit of the standard curve ^36, 37^. Therefore, we use a loess normalisation method for standardising data between assays, which attempts to maintain units in MFI values while also accounting for dilution-dependent variability in signal (i.e., magnitude of between plate variation that differs by MFI range). The robustness of this procedure, however, should be validated with additional laboratory data. For example, repeat testing of one set of endemic sera across multiple plates to simulate assay variability can be used to estimate residual between-plate variance after data normalisation and to determine if the variance is antigen specific. An assessment of potential overfitting using loess compared to linear regression or other normalisation methods should also be explored. It is also important to note that because normalisation is dependent on the standard curve, and adjustment of data below or above the minimum and maximum MFI values of the positive control titrations should be avoided as it may be difficult to extrapolate the relationship at these extremes (as seen with PfMSP119, Supplementary Figure S9). Therefore, designing standard curves to include titrations matching the range of MFI values expected in the samples being processed is important when using this normalisation method.

The ultimate utility of these assays will be the application to high-throughput processing in endemic country laboratories. Minimal specialist equipment is required aside from a Luminex reader, a magnetic plate, and coupled microspheres and standard ELISA laboratory reagents. Therefore, Luminex-based processing is feasible in a range of endemics settings, from local hospitals with back-up electric generators (with consistent supply for at least two 90 minutes incubations and a one hour plate reading) to larger laboratories in urban settings. This will likely require further validation of bead stability during field-based transport and storage conditions. To confirm the reproducibility of the assay and data standardisation methods, between laboratory and user variability testing should be explored. The application of these data to epidemiological analysis will also need to consider antigen-specific kinetics across different endemic settings, in order to validate the use of novel serological markers of recent malaria exposure for routine surveillance or the evaluation of community-based efficacy trials. Standardised methods for determining sero-positivity thresholds for new antigens not previously used should be established.

## Data availability

### Underlying data

Raw data for this study, including output data for antigen and serum sensitivity and stability testing, output data from qSAT assays and data for validation and standardisation, are available on OSF. DOI: https://doi.org/10.17605/OSF.IO/AUJ35 ^32^.

### Extended data

Extended data are available on OSF. DOI: https://doi.org/10.17605/OSF.IO/AUJ35 ^32^.

**Figure S1. Titration of antigen-concentration for bead coupling, across five serum sample dilutions and two positive controls for PfMSP1.19, PfAMA1, and PfGLURP.R2.** Filled circles represent the EC _50_ specific to the serum dilution and positive control. Vertical black line is the median EC _50_ concentration across all serum dilutions and positive controls.

**Figure S2. Titration of antigen-concentration for bead coupling, across five serum sample dilutions and two positive controls for Etramp5.Ag1 and Etramp4.Ag2.** Filled circles represent the EC _50_ specific to the serum dilution and positive control. Vertical black line is the median EC _50_ concentration across all serum dilutions and positive controls.

**Figure S3. Titration of antigen-concentration for bead coupling, across five serum sample dilutions and two positive controls for GEXP18 and HSP40.Ag1.** Filled circles represent the EC _50_ specific to the serum dilution and positive control. Vertical black line is the median EC _50_ concentration across all serum dilutions and positive controls.

**Figure S4. Median fluorescence intensity (MFI) for *Pf*MSP1 _19_ and *Pf*GLURP.R2 of positive and negative samples for each buffer composition.** Buffer compositions tested include buffer A (red), buffer A with *E. coli* lysate (pink), buffer B (blue), and buffer B with *E. coli* lysate (light blue). MFI for positive samples shown in colour (left) and corresponding negatives samples in grey (right) for each buffer composition.

**Figure S5. Median fluorescence intensity (MFI) for Etramp5.Ag1 and Etramp4.Ag2 of positive and negative samples for each buffer composition.** Buffer compositions tested include buffer A (red), buffer A with *E. coli* lysate (pink), buffer B (blue), and buffer B with *E. coli* lysate (light blue). MFI for positive samples shown in colour (left) and corresponding negatives samples in grey (right) for each buffer composition.

**Figure S6. Median fluorescence intensity (MFI) for HSP40.Ag1 and Hyp2 of positive and negative samples for each buffer composition.** Buffer compositions tested include buffer A (red), buffer A with *E. coli* lysate (pink), buffer B (blue), and buffer B with *E. coli* lysate (light blue). MFI for positive samples shown in colour (left) and corresponding negatives samples in grey (right) for each buffer composition.

**Figure S7. Serum sample dilution optimisation.** Mean median fluorescence intensity (MFI) of positive and negative samples tested at four serum sample dilutions (1:100, pink; 1:500, blue; 1:1000, green; 1:2000, purple). Median MFI of negative samples are shown in grey to the right of positive samples shown in colour.

**Figure S8. Levey-Jennings plots for Luminex plate quality control.** Solid points represent the median fluorescence intensity (MFI) values of positive controls, ordered left to right by date of plate processing. Solid horizontal lines represent mean positive control MFI of the reference plates and the dotted lines represent MFI values of either one or two standard deviations from the mean. Coloured lines are the linear regression fit (mean and 95%CI) of change in MFI by date of plate processing, representing estimated signal degradation over a period of 2 months.

**Figure S9. Loess normalisation *Pf*MSP1 _19_ and *Pf*GLURP.R2.** Loess normalisation of antigens *Pf*MSP1 _19_ and *Pf*GLURP.R2 is illustrated for one example plate based on cross-sectional samples from The Gambia. Upper two panels show the loess fit (red) and linear fit (blue) of ∆ MFI on the y-axis (the difference in unadjusted MFI of the standard curve of a single plate and the mean MFI of all standard curves from ten reference plates) and mean MFI on the x-axis. Lower two panels show the raw MFI values of individual samples on the x-axis versus normalised MFI values on the y-axis, and the equality line is shown diagonally in red.

**Figure S10. Loess normalisation Etramp5.Ag1 and Etramp4.Ag2.** Loess normalisation of antigens Etramp5.Ag1 and Etramp4.Ag2 is illustrated for one example plate based on cross-sectional samples from The Gambia. Upper two panels show the loess fit (red) and linear fit (blue) of ∆ MFI on the y-axis (the difference in unadjusted MFI of the standard curve of a single plate and the mean MFI of all standard curves from ten reference plates) and mean MFI on the x-axis. Lower two panels show the raw MFI values of individual samples on the x-axis versus normalised MFI values on the y-axis, and the equality line is shown diagonally in red.

**Figure S11. Loess normalisation HSP40.Ag1 and Hyp2.** Loess normalisation of antigens HSP40.Ag1 and Hyp2 is illustrated for one example plate based on cross-sectional samples from The Gambia. Upper two panels show the loess fit (red) and linear fit (blue) of ∆ MFI on the y-axis (the difference in unadjusted MFI of the standard curve of a single plate and the mean MFI of all standard curves from ten reference plates) and mean MFI on the x-axis. Lower two panels show the raw MFI values of individual samples on the x-axis versus normalised MFI values on the y-axis, and the equality line is shown diagonally in red.
