# Supplementary material for: Optimisation and standardisation of a multiplex immunoassay of diverse Plasmodium falciparum antigens to assess changes in malaria transmission using sero-epidemiology
Source: Wellcome Open Res. 2020 Apr 23;4:26. Originally published 2019 Feb 8. [Version 2] doi: 10.12688/wellcomeopenres.14950.2 (PMC7255915; doi:10.12688/wellcomeopenres.14950.2)
Supplement: Peer review history

# Peer review: Reviewer response for version 2

**DOI:** 10.21956/wellcomeopenres.17149.r38511

**Published:** 2020-05-27

**Authors:** Eric Rogier

**Affiliations:** 1 Malaria Branch, Centers for Disease Control and Prevention, Atlanta, GA, USA

I have been able to re-review this article, and I find the authors’ revisions to be appropriate.

Is the rationale for developing the new method (or application) clearly explained?

Yes

Is the description of the method technically sound?

Yes

Are the conclusions about the method and its performance adequately supported by the findings presented in the article?

Yes

If any results are presented, are all the source data underlying the results available to ensure full reproducibility?

Yes

Are sufficient details provided to allow replication of the method development and its use by others?

Partly

Reviewer Expertise:

NA

I confirm that I have read this submission and believe that I have an appropriate level of expertise to confirm that it is of an acceptable scientific standard.

# Peer review: Reviewer response for version 2

**DOI:** 10.21956/wellcomeopenres.17149.r38510

**Published:** 2020-04-27

**Authors:** Takafumi Tsuboi

**Affiliations:** 1 Division of Malaria Research, Proteo-Science Center, Ehime University, Matsuyama, Ehime 790-8577, Japan

All of my comments have been appropriately addressed.

Is the rationale for developing the new method (or application) clearly explained?

Yes

Is the description of the method technically sound?

Yes

Are the conclusions about the method and its performance adequately supported by the findings presented in the article?

Yes

If any results are presented, are all the source data underlying the results available to ensure full reproducibility?

Yes

Are sufficient details provided to allow replication of the method development and its use by others?

Partly

Reviewer Expertise:

NA

I confirm that I have read this submission and believe that I have an appropriate level of expertise to confirm that it is of an acceptable scientific standard.

# Peer review: Reviewer response for version 1

**DOI:** 10.21956/wellcomeopenres.16307.r35431

**Published:** 2019-05-16

**Authors:** Takafumi Tsuboi

**Affiliations:** 1 Division of Malaria Research, Proteo-Science Center, Ehime University, Matsuyama, Ehime 790-8577, Japan

Antibody responses have been used to characterize transmission and exposure history in malaria-endemic settings for over a decade. The authors are one of the leading groups on such studies, especially *P. falciparum* seroepidemiology. In this study, they carefully validated their Luminex-based antibody measurement system using eight *P. falciparum* recombinant antigens, associated with long- and short-lived antibody responses. They finally standardized antigen-specific bead coupling concentrations, buffer compositions, storage conditions, limits of quantifications and normalization strategies. The results in this work will be very useful for the current and future expansion of the inclusion of new antigens in this Luminex-based platform. I have a few comments that need to be considered:

 1. For which field setting can the MAGPIX platform be applicable (Target Product Profile)? For example, a local hospital with back-up electric generator or a bigger laboratory in the central city.

 2. 6 out of 8 antigens used in this study were expressed as GST-fused recombinant proteins in *E. coli* as shown in Table 1. GST and TT were also included as negative controls. 1) Please clarify how to use MFI on negative controls to normalize MFI on GST-fused antigens. 2) I have another concern about using GST-fused recombinant proteins as antigens for serology because there are many endemic areas of both Schistosomiasis and malaria. If the people had a history or co-infection with this worm, the interpretation of the results will be very difficult. Do the authors have any idea how to overcome this challenge? Therefore, I strongly recommend the authors to use another purification tag such as His-tag in the future development.

Is the rationale for developing the new method (or application) clearly explained?

Yes

Is the description of the method technically sound?

Yes

Are the conclusions about the method and its performance adequately supported by the findings presented in the article?

Yes

If any results are presented, are all the source data underlying the results available to ensure full reproducibility?

Yes

Are sufficient details provided to allow replication of the method development and its use by others?

Partly

Reviewer Expertise:

Malaria protein research, Malaria vaccine research, Malaria molecular biology

I confirm that I have read this submission and believe that I have an appropriate level of expertise to confirm that it is of an acceptable scientific standard, however I have significant reservations, as outlined above.

# Response

**Published:** 2020-04-07

**Authors:** Kevin Tetteh

**Affiliations:** London School of Hygiene & Tropical Medicine, UK

**Reviewer 2**

 We would like to thank Dr Tsuboi for his useful comments

**For which field setting can the MAGPIX platform be applicable (Target Product Profile)? For example, a local hospital with back-up electric generator or a bigger laboratory in the central city.**

*Together with our international colleagues we have demonstrated the utility of the MagPix platform in a number of different settings as evidenced in the following publications (Surendra H* *et al. BMC Med. 2020 Jan 28;18(1):9. doi: 10.1186/s12916-019-1482-7. [* *PMID: 31987052]; van den Hoogen Sci Rep. 2020 Jan 24;10(1):1135. doi: 10.1038/s41598-020-57876-0. [* *PMID: 31980693]; Achan J et al. Clin Infect Dis. 2019 Aug 12. pii: ciz740. doi: 10.1093/cid/ciz740. [* *PMID: 31980693]). We and others have shown that the platform can be used in a wide range of study sites as long as there is access to an uninterrupted power supply. Apart from the Magpix reader, magnetic plate and coupled microspheres, no additional specialist equipment is required, outside of typical laboratory materials.*

*“Minimal specialist equipment is required aside from a Luminex reader, a magnetic plate, and coupled microspheres and standard ELISA laboratory reagents. Therefore, Luminex-based processing is feasible in a range of endemics settings, from local hospitals with back-up electric generators (with consistent supply for at least two 90 minutes incubations and a one hour plate reading) to larger laboratories in urban settings.”*

**6 out of 8 antigens used in this study were expressed as GST-fused recombinant proteins in *E. coli* as shown in Table 1. GST and TT were also included as negative controls. 1) Please clarify how to use MFI on negative controls to normalize MFI on GST-fused antigens. 2) I have another concern about using GST-fused recombinant proteins as antigens for serology because there are many endemic areas of both Schistosomiasis and malaria. If the people had a history or co-infection with this worm, the interpretation of the results will be very difficult. Do the authors have any idea how to overcome this challenge? Therefore, I strongly recommend the authors to use another purification tag such as His-tag in the future development.**

- *Tetanus toxoid (TT) and GST are not intended as ‘negative controls’. TT was included as an internal positive control as it is* *expected that most samples, including malaria endemic and naïve controls, will have measurable MFI values against the TT coupled beads and therefore confirm that the assay was run successfully.* *GST only responses are measured and subtracted from target responses to account for any possible non-specific GST reactivity in samples.*
- *We understand the reviewer’s concern. The GST-tag is based on a glutathione s-transferase from Schistosoma mansoni, which is prevalent in some of the malaria endemic sites under test. However, we and others have found that subtracting the non-specific, or potentially cross-reactive responses to the fusion tag has been demonstrated elsewhere to be effect in measuring antigen specific responses (Dobaño C et al J Infect Dis 2008 [PMID: 18260767]; Ahlborg N et al Clin Exp Immunol 2002 [PMID: 12165089]). That said, we are currently exploring other expression platforms including his-tag to eliminate this concern in the future. *

# Peer review: Reviewer response for version 1

**DOI:** 10.21956/wellcomeopenres.16307.r34827

**Published:** 2019-02-20

**Authors:** Eric Rogier

**Affiliations:** 1 Malaria Branch, Centers for Disease Control and Prevention, Atlanta, GA, USA

The authors describe comparisons of immunoassay conditions utilizing a bead-based system as applied to a *Plasmodium falciparum *8-antigen panel. Data is provided for how antigen coupling concentrations are selected, differences in sample dilution and blocking buffers, and coupled bead storage conditions. Additionally, the authors outline a normalization scheme when multiple assay plates are run for a study. The report is timely, as multiple malaria groups are now using bead-based multiplex assays and investigating new antigens to augment panels. The authors could provide some additional information regarding the details of their assay and reporting, as well as make some text in the manuscript clearer for the inexperienced reader. Furthermore, some figures currently show data for all 8 Pf antigens, whereas others only display data for 2 or 3. At the least, the data for all 8 antigens should be included as Supplementary data.

   

Minor suggestions:

1. Are references available for more detailed information of recombinant antigen production for the 8 presented here (or similar)?
2. Explain in Methods how the beads were washed.
3. Provide detail about GST and tetanus toxoid couplings. Explain what is meant by: “to allow for correction of any GST-specific immunoglobulin”.
4. Explain how “clarified *E. coli* lysate” was produced.
5. Are MFI values reported or MFI-background values?
6. The current flowchart in Figure 1 is a little difficult to read, especially for someone unfamiliar with this assay. It is suggested to add some more text to the boxes to clarify and read more like a protocol. For example, one box reads: “8 uL coupled microspheres, 5 mL buffer A, (1,000 beads per well)”, and would be clearer with: “8 uL of coupled beads added to 5 mL buffer A (for a concentration of approximately 1,000 beads per well)”.
7. Titration curves in Figure 2 for the other antigens would be helpful in Supplementary.
8. Unfortunately, in Figure 3, the authors do not include a storage condition of 4 degrees which most researchers will choose to keep their coupled beads at for practical storage. It’s not clear why conditions starting at room temperature and increasing were chosen, as this wouldn’t be utilized by a group having MAGPIX technology. Even an interruption of cold-chain would only be for a short period of time for this type of work.  
9. For the Buffer Composition comparisons, the authors only offer data for two Pf antigens in Figure 4 and Supplementary Table 4. Please provide this same comparison for all 8 Pf antigens as well as the two control proteins. The same for Figure 6 in that all antigens should at least be shown in Supplementary.  

Is the rationale for developing the new method (or application) clearly explained?

Yes

Is the description of the method technically sound?

Yes

Are the conclusions about the method and its performance adequately supported by the findings presented in the article?

Yes

If any results are presented, are all the source data underlying the results available to ensure full reproducibility?

Yes

Are sufficient details provided to allow replication of the method development and its use by others?

Partly

Reviewer Expertise:

Malaria serological assays

I confirm that I have read this submission and believe that I have an appropriate level of expertise to confirm that it is of an acceptable scientific standard, however I have significant reservations, as outlined above.

# Response

**Published:** 2020-04-07

**Authors:** Kevin Tetteh

**Affiliations:** London School of Hygiene & Tropical Medicine, UK

**Reviewer 1**

We would like to thank the reviewer for their useful comments:

**Are references available for more detailed information of recombinant antigen production for the 8 presented here (or similar)?**

*With the exception of AMA1, the remaining antigens described in the manuscript were generated following methods described in the manuscript and elsewhere (Herman et al. PLoS Negl Trop Dis. 2018 Jun 14;12(6):e0006457. doi: 10.1371/journal.pntd.0006457. [PMID: 29902183]; Tetteh KK et al. Infect Immun. 2013 Oct;81(10):3835-42. doi: 10.1128/IAI.00301-13. [PMID: 23897617]; Polley SD et al. Infect Immun. 2003 Apr;71(4):1833-42. [PMID: 12654798]). The text has been adjusted to reflect this:*

*“Each antigen was generated and expressed in Escherichia coli as glutathione S-transferase (GST)-tagged fusion proteins using methods as described elsewhere (PMID: 29902183, PMID: 23897617, PMID: 12654798). The exception to the panel was PfAMA1, which was expressed in Pichia pastoris as a histidine-tagged protein (PMID: 17192270).”*

**Explain in Methods how the beads were washed.**

*The following sentences have been included into the qSAT procedure to clarify the wash step.*

*“Next, 50 μl of this combined microsphere mixture was added to a 96-well flat bottom plate (BioPlex Pro™, Bio-Rad Laboratories, UK) and washed once by placing the assay plate onto a magnetic plate separator (Bio-Plex®, Bio-Rad Laboratories, UK) and incubated for 2 minutes at room temperature. Plates were then inverted forcefully to remove the liquid and 100 μl of PBS-T (1xPBS, 0.05% Tween-20) added to each well. “*

**Provide detail about GST and tetanus toxoid couplings. Explain what is meant by: “to allow for correction of any GST-specific immunoglobulin”.**

*Tetanus toxoid (TT) coupled beads were included as the Tetanus vaccine is widely used and therefore useful as an internal control. It would be expected that most samples, including malaria endemic and naïve controls will have measurable MFI values against the TT coupled beads and therefore confirm that the assay was run successfully. This is a particularly useful control when samples are assayed as single wells (with no duplicate result to compare to).*

*The GST-tag used in the purification of the recombinant proteins is based on a glutathione s-transferase from Schistosoma mansoni, a blood trematode common to some of the malaria endemic sites under test. As several of the antigens are expressed as GST-tagged fusion proteins it is important to correct for any background, or non-malaria specific reactivity to the GST-tag. Assaying for reactivity to beads coupled with purified GST allows us to measure the level of reactivity to the tag, if any, and background correct the response by subtracting the GST response. Subtraction of non-specific, or potentially cross-reactive responses to fusion tags in serological studies has been demonstrated elsewhere (Dobaño C et al J Infect Dis 2008 [PMID: 18260767]; Ahlborg N et al Clin Exp Immunol 2002 [PMID: 12165089])*

**Explain how “clarified *E. coli* lysate” was produced.**

*To produce the bacterial lysate, untransformed E.coli were cultured using the Studier recipe for autoinduction media under the same conditions as used for expression of the recombinant protein with one exception. The addition of an antibiotic for selection of recombinants is omitted. After culturing for approximately 18 hours a* *t 37 ^0^C, the cells are harvested by centrifugation, resuspended in PBS and lysed using a high pressure homogeniser. The lysed material is then centrifuged at 16,000 rpm for 15 min to pellet the lysed E.coli membranes. The clarified lysate is then aliquoted and stored at -20 ^0^C until needed.*

*As sentence has been included in the text to summarise this point:*

*“Bacterial lysate was generated from the culture of untransformed E.coli and used in the preparation of assay buffers as non-specific protein to eliminate background reactivity to E.coli proteins (PMID: 26216993).”*

**Are MFI values reported or MFI-background values?**

*All results are reported as MFI-background and the following sentence has been added to the methods: “All data reported and analysed are in units of background subtracted MFI.”*

**The current flowchart in Figure 1 is a little difficult to read, especially for someone unfamiliar with this assay. It is suggested to add some more text to the boxes to clarify and read more like a protocol. For example, one box reads: “8 uL coupled microspheres, 5 mL buffer A, (1,000 beads per well)”, and would be clearer with: “8 uL of coupled beads added to 5 mL buffer A (for a concentration of approximately 1,000 beads per well)”.**

*We appreciate the reviewer’s comments and the flowchart has been edited accordingly and a new version uploaded.*

**

**Titration curves in Figure 2 for the other antigens would be helpful in Supplementary.**

*The titrations curves for the remaining antigens were previously included in the supplement (Figures S1,S2, and S3)*

**

**Unfortunately, in Figure 3, the authors do not include a storage condition of 4 degrees which most researchers will choose to keep their coupled beads at for practical storage. It’s not clear why conditions starting at room temperature and increasing were chosen, as this wouldn’t be utilized by a group having MAGPIX technology. Even an interruption of cold-chain would only be for a short period of time for this type of work**

*Coupled and uncoupled beads are stored at 4 ^0^C as standard. However, coupled beads are routinely shipped globally to collaborator sites, where disruptions to the cold chain can and has occurred. As such we felt it was necessary to evaluate the potential impact of suboptimal shipping and/or storage conditions on the functionality of the coupled beads. The following text has been included in the discussion as a point to consider in future studies: “Temperatures of 22°C and above were tested in this study to assess the potential impact of disrupted cold chain during shipping to or long-term storage in laboratories in low- and middle-income settings. Further work should also investigate bead stability during long-term storage at 4°C, the standard storage condition for most research groups using Luminex platforms.”*

**For the Buffer Composition comparisons, the authors only offer data for two Pf antigens in Figure 4 and Supplementary Table 4. Please provide this same comparison for all 8 Pf antigens as well as the two control proteins. The same for Figure 6 in that all antigens should at least be shown in Supplementary.**

*This has now been included in Supplementary data as Figures S4-6 for buffer testing, and Figures S9-11 for loess normalisation*
